# Stroke survivors’ experiences of team support along their recovery continuum

**DOI:** 10.1186/s12913-019-4533-z

**Published:** 2019-10-21

**Authors:** W. Hartford, S. Lear, L. Nimmon

**Affiliations:** 1 Centre for Health Education Scholarship, Faculty of Medicine, P. A. Woodward Instructional Resources Centre (IRC), University of British Columbia, 429-2194 Health Sciences Mall, Vancouver, B.C V6T 1Z3 Canada; 2 Faculty of Health Sciences, Simon Fraser University, Blusson Hall, Room 11300, 8888 University Drive, Burnaby, B.C V5A 1S6 Canada; 3 Department of Occupational Science and Occupational Therapy, Faculty of Medicine, University of British Columbia, 2211 Wesbrook Mall T325, Vancouver, B.C V6T 2B5 Canada

**Keywords:** Stroke rehabilitation, Collaboration, Healthcare inequalities, Empowerment, Health services structures

## Abstract

**Background:**

A coordinated stroke rehabilitation care team is considered optimal for supporting stroke survivors from diagnosis to recovery. Despite this recognition, many stroke survivors cannot access essential rehabilitation services. Furthermore, there is a lack of understanding of stroke patients’ and their caregivers’ rehabilitation needs and wishes. We sought to gain insight into healthcare and social structures from the perspective of patients and caregivers that can better support long-term stroke recovery.

**Methods:**

We conducted individual interviews with 24 participants comprised of stroke survivors, spousal caregivers, stroke support group coordinators, and speech pathologist. Participants were recruited through three stroke survivor support groups. An empowerment lens was integrated into data analysis and data interpretation.

**Results:**

Two dominant themes captured participants’ experiences through stroke survivors’ trajectory of care. 1) Experiences of managing stroke. This theme identified stroke survivors and spousal caregivers’ experiences with stroke recovery, rehabilitation, and fulfilling unmet needs. 2) Resources of support. This theme described the social and financial support structures drawn upon to assist with stroke rehabilitation.

**Conclusions:**

The study highlighted a lack of teamwork between stroke survivors, spousal caregivers, and health professionals. This fragmented care was compounded by inequities in rehabilitation programs and health services resulting in what appeared to be a disempowering rehabilitation process. Although stroke recovery groups were a significant source of support for stroke survivors and spousal caregivers, participants perceived they were overlooked, by stroke recovery healthcare providers, as a site for stroke recovery healthcare services. An empowerment approach to stroke rehabilitation involves collaboration between stroke survivors, caregivers, healthcare providers, health services, and existing community stroke support structures. Framing stroke based care through an empowerment lens may serve to address stroke rehabilitation inadequacies and inequities.

## Background

Stroke is a leading cause of death in adults [1–6] and the most prevalent cause of disability globally [1–3, 7–14]. Rehabilitation, the process which enables individuals to regain physical, intellectual, psychological, sensory, and social functionality [3, 8, 15] is key to acute, and short and long term recovery outcomes [3, 15]. A coordinated stroke rehabilitation and recovery care team is considered the optimum way to address individuals’ needs and provide stroke survivors with satisfactory care from diagnosis to recovery in the acute phase and in the long term [4, 10, 12, 15].

The 2015 Canadian Best Practices Guidelines, an evidence-based report, strongly support stroke survivor and informal caregiver access to a professional rehabilitation team across the recovery continuum [15]. Stroke care teams should include specially trained physicians, physiatrists, neurologists, physiotherapists, occupational therapists, speech-language pathologists, nurses, social workers and dieticians [15]. Pharmacists, case managers, psychologists, palliative care specialists, recreation and vocational therapists, and stroke recovery group liaisons may also be included on the team [15]. Furthermore, the stroke survivor and family are also included in this concept of stroke care team [15]. Diverse members of stroke care interprofessional teams are encouraged to work closely and supportively to fulfil stroke survivor needs and goals, and to improve recovery outcomes [12, 15]. In addition to acute and inpatient stroke rehabilitation and recovery care, these recommendations also highlight the importance of coordinated outpatient and/or community based rehabilitation [15]. Such services include day hospital programs, community and/or home based rehabilitation programs, and community recreational programs [15].

While there have been significant efforts in stroke patient acute care and short term in-patient rehabilitation there has been a lack of attention paid to long term care post stroke [10, 16]. Continued therapy and support after early stroke treatment and discharge have been shown to improve functional status well-being beyond 6 months [1, 3, 15] and improve recovery outcomes for stroke survivor and their caregivers [1, 10, 12]. However, once discharged from institutional care, many stroke survivors cannot access essential rehabilitation services such as physiotherapy, occupational therapy, homecare, and speech therapy [7, 11, 15, 16].

Lack of community care and limited support from primary healthcare and community services contribute to a perception of marginalization and abandonment of stroke survivors and caregivers following hospital discharge [6, 16]. Given the anticipated increase in stroke patients due to an aging population [8, 11, 12, 14, 17], there is a growing need for adequate long-term community care following discharge for patients [10, 16]. However, we lack understanding of stroke recovery from the perspective of stroke survivors and their informal caregivers (family members, friends, and community members) [2, 17, 18], and their requirements for long-term stroke rehabilitation [7, 16, 18]. Without this vital understanding we have little insight into what form models of stroke care should follow [10, 18, 19]. Filling these gaps may provide health systems and healthcare providers with a better understanding of the requirements for robust long term stroke rehabilitation. The purpose of this study was therefore to investigate rehabilitation experiences of stroke survivors and their informal caregivers. The aim was to identify healthcare and social structures which can better support long-term stroke recovery.

### Empowerment theory

An empowerment lens was employed to sensitize our analysis of the data as it has the potential to facilitate the identification of processes that support and influence stroke management outcomes. Empowerment theory considers processes, such as social structures and mechanisms, which enable individuals, organizations, or communities to gain control (an empowerment outcome) of issues that affect their lives [20–22]: for instance, gaining access to available resources [21]. Empowerment is a multidimensional relational construct which occurs within and between individuals, organizations, communities, and/or the sociopolitical environment [21–23], and is developed through collaborative partnerships [20, 22]. Psychological empowerment processes provide individuals with a means to gain control [21] or mastery [23] over their lives.

The concept of psychological empowerment is not new to chronic healthcare management, rehabilitation, and/or long term health management [24, 25]. Empowerment healthcare frameworks have been used to promote collaboration and equity within healthcare interactions which enable self-management of long term chronic conditions [25, 26]. From a stroke recovery and rehabilitation perspective, social structures and mechanisms may include: acute and post-acute care, inpatient and outpatient rehabilitation, community stroke support programs and clinics, and multidisciplinary stroke rehabilitation healthcare teams [15, 17]. Healthcare system rules, regulations, and protocols, such as rehabilitation assessment criteria, may also contribute to stroke care social structures and mechanisms. For example, criteria which determine stroke care include acute/recent stroke, medical stability, readiness to participate, cognitive ability to participate, and ability to access community care [15]. Psychological empowerment mechanisms also include personal attributes such as development of coping skills, self-efficacy, self-sufficiency [20, 22, 23].

Stroke rehabilitation itself may be considered an empowering process. For example, Peoples [5] reports that stroke rehabilitation enabled some stroke survivors to gain coping skills, meet their physical and non-physical needs, collaborate with professionals, and take control and assume responsibility for their care. Hewitt et al. [12] identified “efficient, open, and equitable communication”, and “collaboration and coordination” between stroke survivors/caregivers and healthcare professionals as key mechanisms for effective stroke rehabilitation [12. p.335–336].

Finally, patients who are empowered report better health and wellbeing outcomes [24–26]. Bravo [26] reports improved adaptation to condition, quality of life, satisfaction with life, and healthcare independence in patients who scored higher on an empowerment measurement scale. Therefore, investigating participant experiences from an empowerment perspective draws our attention to structures and mechanisms that support individual empowerment and improve stroke management outcomes for stroke survivors and their informal caregivers.

## Methods

### Research questions

How do stroke survivors and their network of care (healthcare professionals and informal caregivers) experience team support throughout the continuum of stroke recovery? What social structures would empower stroke survivors to meet their self-management needs?

### Study design

This study took place in a major city in Western Canada. Human ethics permission was granted to interview stroke survivors and caregivers (such as spouses, stroke recovery group leaders, and therapy providers). Two researchers, LN (a social scientist) and WH (an adult educator) conducted the research project. The objective of this study was to explore experiences of stroke care throughout the continuum of stroke recovery. Therefore, a qualitative descriptive design was used [27] to elicit participants’ descriptions of their stroke recovery experiences within the Canadian healthcare system. Canada has a universal healthcare scheme (Medicare) which provides access for all Canadian residents to medically-necessary hospital and physician services [28]. Medicare funding is provided by Federal, Provincial, and Territorial governments. Health insurance plans are the responsibility of Provincial and Territorial governments [28]. Allied health and alternative therapy services coverage are, in general, not funded in this Western Canadian province [29]. Extended healthcare plans, which may fully or partially cover additional services, are available through employment schemes or as individuals. Individuals without extended healthcare pay in full for additional services.

### Participants and sampling strategy

The researchers sought a sample size to capture various multifaceted perspectives of stroke recovery [30]. In addition, the researchers used a combination of purposive and convenience sampling to recruit participants from locations where participants of interest (stroke survivors and various caregivers) would be present in sufficient numbers [30]. Information, provided by a city stroke rehabilitation centre, indicated that community stroke support groups may be an appropriate recruitment site for prospective participants. The anticipated sample size for stroke survivors for this study was 12–15 which the researchers expected would be adequate to reach theoretical sufficiency [31]. The sample size for spousal caregivers was determined by caregivers who were present with stroke survivor partners who consented to be interviewed. The sample size of other caregivers, identified as coordinators or therapists, was determined by their availability at the stroke rehabilitation groups.

### Recruitment

The research project was presented to three stroke recovery groups by LN and WH. WH followed up with stroke survivors and caregivers who indicated an interest in the study. Prospective participants were provided with consent packages which provided details of the study, interview process, and consent form. The packages were delivered by e-mail, post, and by hand. Interviews were scheduled with participants who met the inclusion criteria and who provided written consent to participate in an audio-recorded interview.

### Data collection

The interviews were conducted between September 2015 and February 2016. The majority of the interviews took place on the premises where the recovery groups held their meetings to accommodate the patients’ limitations (wheelchair access and ability to travel to the interview location). At all facilities a private room was provided for interviews. Three interviews were conducted in participants’ homes and one interview in a hospital cafeteria. WH conducted face-to-face interviews with all 24 participants.

The researchers developed a semi-structured interview guide (Additional file 2: Interview guide) to explore participants’ experiences with stroke rehabilitation in the community. Questions were developed to identify how patients’ preferences, goals, and values were considered in their treatment decisions. The researches also explored concepts of empowerment, autonomy, power, and agency and control in interactions with healthcare providers using open ended questions. Demographic questionnaires were also completed (Additional file 1: Demographic questionnaire). As data collection progressed, interview questions were refined to gather insights into emerging concepts or to inquire about a critical area, such as identifying members of an individuals’ healthcare team. This flexible design is typical for qualitative research [30]. Teamwork as captured in the interview guide was conceptualized as including the stroke survivor, informal caregivers, and healthcare professionals. Specific questions pertained to treatment goals, communication and interaction between healthcare team members, healthcare team support, role on the healthcare team, and experiences of empowerment. All interviews were audio-recorded. Interviews ranged in length from 17 min to 1 h and 32 min. The majority of interviews were in the range of 30–40 min.

In addition to the interviews WH took field notes of each interview. These notes documented her observations, emotions, and reflections of the interviews. Observations of interest included participants’ mobility, behaviour, interactions with their partner if present. Additionally, field notes captured how the interview proceeded and the contextual environment where data collection took place (e.g. the ambience and privacy of the interview room). In total 22 pages of reflective field notes were written. Furthermore, the researchers employed the concept of data saturation and collection of rich and thick data [32]. When no new data, themes, or coding arose from the analysis, the researchers considered the quality and quantity of the data against the research questions and analytical lens [32]. Data collection ceased when a robust understanding of the phenomenon emerged in analysis.

### Data analysis

The researchers employed several strategies to ensure methodological rigor: validity, reliability, authenticity, and credibility throughout the data collection and analyses processes. Firstly, the use of semi-structured interviews, as described above, enabled the researchers to return to, and critically reflect on the research questions and purpose of the study. This stimulated consistency and reliability in the interview protocol while at the same time allowing for identification of different and new descriptions of experiences [30]. Secondly, field notes compiled immediately after each interview by WH provided a documented contextual account of each interview. These accounts provided a basis for discussion with LN about the context of the interview and assisted in identifying researcher bias. Thirdly, all interviews were audio-recorded and transcribed verbatim. Participants were provided with the opportunity to review the transcript of their interview. These three strategies for data collection provided opportunities to cross-check the data against the research questions for reliability, authenticity, and credibility [27]. The interviews took place on different days and at different locations, thus the field notes served to identify nuances, such as the influence of the interview environment (privacy, ambiance, and accessibility), that may influence dependability of the data [27]. Fourthly, the researchers have provided a rich description of the theoretical framework, and data collection and analysis to allow for the potential for transferability to other similar populations [27]. Integrating concepts of validity and reliability to the data analysis, WH and LN organised the data, coded, analysed, and searched for alternative interpretations [27]. The data analysis process is outlined in detail below.

The audio-recorded interviews were transcribed, coded, and analysed concurrently by LN and WH using NVivo 11 QSR International. We employed a three stage qualitative analysis approach to facilitate the exploration of participants’ experiences of managing stroke as described by LeCompte and Schensul [30]. This inductive analysis involved item analysis which generated conceptual statements concerning patterns of relationships in the data which produced general insights into our topic of interest. Initially WH examined the data and grouped items of interest as primary codes for data organization [30]. In addition to grouping similar items, dissimilar or competing items were sought to reduce researcher bias and premature judgements. Approximately 50 codes were generated such as “patient advocates for self”, “ageism”, and “being heard or unheard”. As the item analysis progressed, WH observed that participants experienced difficulties having their needs fulfilled in relation to attaining what they (stroke survivors and spouses) perceived to be their full recovery potential. It began to emerge that these stroke survivors and their informal caregivers were not well supported by the healthcare system. Throughout item analysis WH and LN discussed and refined the codes and developed pattern codes (e.g. adapting to stroke, unmet needs, and fulfilling unmet needs) in a reflective iterative process [30]. Noticing the sentiment of a lack of support made by patients and caregivers, WH drew on an empowerment lens for the third stage of analysis (structural analysis) which identified broad theoretical themes such as socioeconomic differences, treatment inequities, and support mechanisms. At this time, review was sought by WH and LN from the other author SL to enhance this reflective analytical process and enhance research credibility.

## Results

A total of 24 participants were recruited: 16 stroke survivors (Female = 5, Male =11 aged 48–87), 4 spouses, (female aged 62–80), 3 stroke recovery group co-ordinators (female), and 1 speech pathologist (female). Of the stroke survivors 9 were married or in a common law relationship with partners who assisted daily with their care, 4 were widowed, 1 was separated, 1 was single, and 1 was divorced. See Table 1 (Stroke survivor demographic information) and Table 2 (Spousal caregiver demographic information) which present demographic data for stroke survivors and spousal caregivers.

**Table 1:** Stroke survivor demographic information

Age (years) at time of interview	Marital status	Duration of stroke	Work status	Extended medical coverage
Range = 48–87 Mean = 68.75	Married/common law = 9 Single = 1 Widowed = 4 Separated /divorced = 2	Range = 3 months – 26 years Mean = 8.74 years	Unemployed = 6 Retired = 8 Volunteer = 1 Employed = 1	Yes = 5 No = 6 Private funds = 5

**Table 2:** Spousal caregiver demographic information

Caregiver	Age/years	Work status	Interview
*N* = 4 = Female	Range = 62–80 Mean = 73.5	Retired = 3 Employed =1	With partner =2 Without partner =2

In general, participants described how stroke recovery played out as a trajectory of adapting to a new way of living day-to-day. Julie (speech pathologist) reported that coming to terms with the effects of stroke took time with stroke survivors and their caregivers often experiencing a sense of loss and grief. Stroke survivors described how they had to start life again “from zero” and relearn how to walk, speak, bathe, dress themselves, learn to use public transport, and make new living arrangement to accommodate the physical consequences of stroke. Other participants described emotional reactions to stroke recovery. Gwen (spouse) described how not knowing about stroke had been both “scary” and “frustrating”, but while her fears had subsided she remained frustrated with the healthcare system. Tony (stroke survivor) questioned whether survival was worthwhile as “living in this way is not really living”, and Christine (spouse) described feeling guilty that she had not been able to address her stroke survivor spouse’s needs concluding:

it’s a very difficult health problem because strokes are personal. They’re all different. Everybody who has a stroke has different symptoms, different progress, different needs. And it’s a search for the caregiver to find what might work. And it isn’t that people can’t tell you. It’s just that they don’t know because the stroke is so individual.

Stroke survivors reported different rehabilitation treatments, therapies, rates of recovery, and degrees of functionality. Rehabilitation programs and discharge procedures differed by institution, timing, and availability of and access to rehabilitation resources and services. Despite these idiosyncratic differences, two dominant themes captured the main essence of the findings: 1). Experiences of managing stroke, and 2). Resources of support. All participants are assigned a pseudonym to maintain confidentiality. A summary of the themes is presented in Table 3.

**Table 3:** Results: Summary of themes

Themes	Sub themes
***Theme 1: Experiences of managing stroke*** Participants described how stroke recovery played out as a trajectory that included a process of adaption to a new way of living day-to-day. Many participants reported how their rehabilitation needs were not readily met by the healthcare system. Their process of recovery was therefore hindered by a sense of being left to their own devices to access resources.	***Unmet needs*** Participants described the many ways in which stroke survivors’ and caregivers’ goals for recovery, and rehabilitation needs were not met. ***Fulfilling unmet needs*** Participants described how they circumvented barriers to acquiring appropriate rehabilitation that fulfilled their unmet needs.
***Theme 2: Resources of support*** Participants described their various sources of financial and societal support.	***Financial support*** Participants described how their socioeconomic status significantly influenced their ability to obtain the rehabilitation services and support they perceived they needed to meet their goals. ***Social support*** Participants described how social support was a significant positive influence on stroke rehabilitation for stroke survivor and spousal caregivers.

### Theme 1: experiences of managing stroke

This major theme generated two sub-themes that related to experiences managing stroke: describing unmet needs, and finding ways to fulfill those unmet needs. Participants reported how recovery was hindered as their rehabilitation needs were not readily met by the healthcare system. Consequently they were left to their own devices to access resources.

#### Unmet needs

Throughout the participants’ descriptions of stroke recovery experiences was a narrative thread of unmet needs, inequitable access to resources, and a sense of frustration with the healthcare system. Participants described two in-patient recovery facilities (IRFs) with the larger facility perceived as the IRF of preference due to the affordances and reputation of the facility for intensive therapy. The smaller facility was described as offering fewer, and poorly coordinated opportunities for intensive therapy. Several stroke survivors and caregivers expressed dissatisfaction most often when a preferred treatment or rehabilitation program was denied due to the stroke survivor’s age or perceived lack of potential to improve.

Because you see he’s too old for [larger] rehabilitation centre… but they won’t take people over 65, which I think is wrong…But I always had the feeling that, the minute they found out George was 86 they sort of turned off the emergency button. And I didn’t like that. (Jennifer, spouse)

Descriptions provided by stroke survivors and caregivers indicated their perceptions of their capabilities, therapeutic needs, and expectations for the future often differed from those of their healthcare providers. Tony (stroke survivor) described being told he had plateaued and he must accept “this is as good as it gets”, and Nicolas’ rehabilitation assessment indicated he would never talk or walk again. Implicit in descriptions of limited opportunities for recovery and unsupportive conversations with some health professionals was a sense of being “written off”:

What was… really disappointing, is they kept on writing him off saying he [Eric stroke survivor] would never speak again. He would never walk again. He would never do this. He would never do that… I just found them to be extremely negative (Gwen, spouse).

Gwen also indicated that while Eric received several rehabilitation assessments their goals for rehabilitation were not taken into account. James (stroke survivor) suggested that healthcare providers, such as physiotherapists, had limited his physical recovery as they tended to rely on test results and theoretical expected progression to determine therapy. This medical information was prioritized over James’ perception of his capabilities and expectations.

Participants also alluded to the limitations of stroke rehabilitation programs. Elizabeth (stroke survivor) and Christine (spouse) both reported that scheduled therapy sessions were often cancelled due to unavailability of rehabilitation staff. Debbie (spouse) suggested that essential intensive therapy was minimal and not prioritized by the healthcare system:

I think it’s the system more than the people, and I think the system just doesn’t work for intensive therapy… I think there’s been a real lack of intensive therapy…. at least for the first three months we needed way more therapy. There was a lot of assessing, therapy minimal at times…frustrating.

#### Fulfilling unmet needs

Stroke survivors expressed a desire to get better and thus persevered despite the many structural barriers they encountered. To obtain the therapy and support they needed stroke survivors and spousal caregivers reported advocating for their preferred rehabilitation program, and organising their own homecare, physiotherapy, speech therapy, exercise opportunities, and other support requirements. Several participants indicated that advocacy and being empowered was part of their role, or responsibility, as a stroke survivor or a caregiver. James (stroke survivor) stressed the importance of having an advocate as he perceived that as a patient he was often not listened to. He reported that his spouse had advocated for more in-patient rehabilitation by refusing to take him home. Elizabeth (stroke survivor) described how she drew on the services of a lawyer to successfully appeal to her strata corporation to make her condominium building wheel chair accessible. Several participants indicated they did not feel empowered regarding stroke treatment and management throughout recovery, while Debbie (spouse) despondently responded that “you have no choice”, implying that you have to take charge.

Participants’ recovery descriptions identified characteristics such as determination, motivation, and perseverance which had help them to regain some of their lost functionality. Nicolas had regained his ability to walk and speak over a period of 6–12 years. Being told by his therapist that there would be no more improvement had motivated Philippe (stroke survivor) to improve his left hand function. Matt’s (stroke survivor) desire to regain speech helped him to persevere with speech therapy, and Bess (stroke survivor) indicated that her assertive and stubborn personality had supported her recovery:

But I’m quite pushy. And I think that my redeeming quality in the recovery from the stroke was bloody-mindedness. It was just-- there was no way I was not going to do it. And so there was a great deal of effort on my part.

Participants also described how they obtained the therapy and care that they perceived they needed from available health services and community resources.

### Theme 2: resources of support

All participants indicated that they drew on a variety of resources for support and described several factors which significantly influenced their ability to meet their perceived needs for managing stroke. Two key resources were identified as financial support and social/community support.

#### Financial support

Our analysis suggested that socioeconomic status significantly impacted stroke management, particularly with respect to fulfilling rehabilitation needs to optimize stroke survivor outcomes. Participants indicated that the cost of stroke rehabilitation could be high and not all stroke survivors were financially equipped to self-fund the rehabilitation mechanisms they considered necessary. While several stroke survivors reported having medical insurance (which covered some or all post discharge rehabilitation therapy requirements) others paid out of pocket for these services.

So because I have good extended health [insurance] I was actually gold-plated, really. When I got home, I was able to hire this physio...And he came to the house once a week and gave me physio for quite a period, several months I suppose. And I had him again recently (Peter, stroke survivor).

However, several stroke survivors were recipients of disability allowance, their only source of income, and were unable to pay privately for services. They relied on limited resources available to them in the healthcare system and local community. Tony (stroke survivor) made use of low cost yoga for other chronic conditions, which he adapted to his needs explaining: “…there was no chance of my being able to afford to go privately to a physiotherapist….Because essentially they want you to then go buy your own services. But who’s in a position to do that on a disability grant?”

Stroke recovery groups also provided low cost access to limited rehabilitation therapy. However, stroke survivor group co-ordinators and the speech pathologist identified lack of funding as a potential barrier to providing rehabilitation services for stroke survivors and caregivers.

Homecare encompassed a range of services such as nursing care, help with bathing, laundry, and food preparation. For example, Sharon (stroke survivor) had the financial means to employ a full time housekeeper who took care of all of these services. However, the majority of stroke survivors where dependent on inadequate and inconsistent homecare services provided by the healthcare system. Vera and Josaia (stroke survivors) received very limited homecare, despite their significant disabilities, which impacted their food preparation, house cleaning, and bathing capabilities.

#### Social support

Social support seemed to make a notable contribution to the recovery and well-being of stroke survivors and spousal caregivers. Most stroke survivors indicated that they were supported by a spouse, other family members, and/or friends. Stroke survivors and spouses also concurred that stroke recovery groups were an important factor for stroke recovery. James (stroke survivor) indicated stroke recovery groups substituted for the lack of rehabilitation discharge follow-up by providing an environment where stroke survivors could obtain therapy services, as well as emotional support. In addition, stroke recovery groups offered a place where stroke survivors could meet, develop new friendships, and rebuild their lives. Participants described stroke recovery groups as nurturing places where members did not feel self-conscious about their physical and cognitive limitations. Tony (stroke survivor) described how stroke survivors could learn from each other which helped in setting goals. For Peter (stroke survivor) “… it’s really the emotional support, the fellowship and the goodwill that you can come here and having had a stroke, everybody else has had a stroke, so you don’t have to explain it to anybody. Everybody understands.”

Stroke recovery groups also provided stroke survivors with access to practical resources: such as community volunteer assistance filing tax returns and opportunities for learning about practical tools to aid recovery. Groups also provided information about community resources: such as homecare services and opportunities for volunteering.

Stroke survivor groups were described as empowering because they were a space where stroke survivors interact, support, and learn from each other. Eve (stoke group co-ordinator) described how leaders and members of the stroke recovery groups collaborated to identify members’ abilities and work with them to further their recovery. She also reported how a member had taken on the role of treasurer and helped her with: (a) typing letters, (b) preparing an earthquake readiness program, and (c) raising money for their group by presenting their program to other stroke survivor groups.

Despite the benefits stroke recovery groups offered members, the stroke recovery group co-ordinators and speech pathologist alluded to limited healthcare system support for the groups in terms of referral.

And very often as hard as we try they don’t even mention that there’s a stroke recovery branch anywhere in their neighbourhood…People find us through word of mouth. They find us from [large] rehabilitation centre or [small] rehabilitation centre if they happen to go there…They [family doctors] often don’t even know what’s available in the community. (Peggy, stroke survivor group co-ordinator)

Stroke survivor group co-ordinators experienced barriers to promoting their groups such as not being allowed to hand out resource pamphlets in stroke units. They expressed frustration about how stroke healthcare appeared to be focused on the acute care setting rather than extended community care. Stroke survivors were also concerned about lack of publicity and reluctance of in-patient rehabilitation facilities to refer stroke survivors due to “privacy issues” (Peter, stroke survivor). Stroke survivor group co-ordinators’ descriptions of past experiences suggested that stroke recovery groups had, in the past, been more connected with the community healthcare system in terms of liaison with hospitals, and interactions with visiting stroke recovery therapists: “There used to be a liaison nurse who was a member of the public health team who worked at the hospital…” (Hannah, stroke survivor group co-ordinator).

## Discussion

Our analysis suggests that the stroke recovery processes that these participants experienced did not encompass an empowerment processes. Rather, the findings highlighted participants’ disempowering experiences arising from inequalities in the healthcare system provision of stroke rehabilitation programs. Furthermore, a lack of collaboration and participation, essential for empowerment [20, 22] and teamwork [7, 12], between stroke survivors/ their informal caregivers and their healthcare providers was identified. There also appeared to be a lack of coordination between community resources, such as stroke recovery groups, and the healthcare system. However, our analysis also suggests that participants, by finding ways to achieve their goals through building social connections and advocating for their needs, became empowered.

### Being empowered

This analysis identified the challenging need for stroke survivors to be empowered: that is, engage in empowerment related mechanisms such as self-help, self-efficacy, self-sufficiency, and collaboration and participation with others [20, 22, 23]. Our findings suggested that stroke survivors and caregivers may require certain characteristics or abilities in order to fulfill their needs. Characteristics included being strong-willed and determined with the ability to identify gaps in their care, seek information and knowledge, advocate, and persevere. Resilience and motivation, along with the cognitive ability to plan, organise, and initiate, are important factors for stroke recovery [14] and transitioning back into the community [33].

Our findings indicated that stroke survivors and caregivers sought knowledge and information, and in various ways had to assume responsibility for their care in their new situation. However, these behaviours were burdensome and related to expressions of disempowerment, hopelessness, and helplessness. Explicit expressions such as “I don’t feel empowered” and “you have to be [empowered]”, and implicit expressions of frustration, fear, guilt, and depression, along with a sense of the need to fight for services, *should* be considered disempowering since empowerment may also be understood by its absence [5, 20]. Absence of empowerment is found in individuals’ loss of control over their life or their perception of being powerless, hopeless, helpless, subordinate, alienated, dependant, victimised, or the object of paternalism [5, 20, 36]. Furthermore, descriptions of being “written off”, unsupportive conversations with health providers, and being told “this is as good as it gets” are interrelated with a sense of hopelessness, helplessness, and disempowerment.

### (dis) empowerment and in-patient stroke rehabilitation

The disparate experiences of rehabilitation services identified in our findings suggest an inequitable healthcare system: one with apparently inflexible mechanisms which denied many stroke survivors and caregivers the holistic treatment and therapeutic support they needed. Equitable access to resources is inherent to the process of empowerment (5, 22). Stroke rehabilitation care is generally determined by medical, cognitive, and mobility factors [12, 17], severity of stroke [33], medical stability, nursing care requirement, therapy requirement, tolerable amount of activity [34], and age [9, 34]. Our analysis suggests limitations with these guidelines. For example, using age to determine rehabilitation needs may deny stroke survivors a preferred or optimal standard of care.

Also inherent to the process of empowerment are collaboration and participation in decision making [5, 22]. Rehabilitation outcomes are improved when individuals actively participate in decision making and engage in proactive behaviours [17, 22]. Contrarily, lack of involvement in rehabilitation decision processes may lead to stroke survivor and caregiver disempowerment and disengagement from the process of recovery [17]. In our study, collaboration between health professionals, stroke survivors, and caregivers was not the norm. Rather, the findings mirror the literature [6, 8, 15, 18] and suggest that stroke survivors and caregivers were given little opportunity to participate in healthcare team decisions about rehabilitation therapy and recovery plans. This lack of engagement may have created a mismatch between stroke survivor/caregiver and healthcare provider goals or expectations for recovery. Typically, health providers’ goals for rehabilitation are for stroke survivors to be able to undertake basic activities of daily living [8]. These goals are often at odds with stroke survivors’ and caregivers’ desire for a return to pre-stroke activities [11, 13, 17]. Rehabilitation structures that do not foster equitable treatment and therapy inhibit opportunities for good holistic care [5], and are unlikely to support development of skills associated with empowerment [22].

### Stroke rehabilitation in the community: the interface between empowerment and disempowerment

Lack of collaborative healthcare system support and inequities in rehabilitation structures were exacerbated following discharge into the community. Mirrored in the literature [6, 18, 35] healthcare system support for the stroke survivors and caregivers in our study was limited, lacked consistency, and was perceived as inadequate, unhelpful, and stressful. In addition, stroke survivor socioeconomic variation had significant implications for equitable access to on-going rehabilitation (homecare, physiotherapy, and speech therapy). Participants reporting extended healthcare benefits, or other financial supports, were better able to access rehabilitation resources than those who were economically constrained. Limited access to rehabilitation services has been reported to limit recovery potential [9], whereas equitable access to resources is found to promote the development of skills such as resilience, self-reliance, and self-governance, which are associated with empowerment [22, 36]. Skill development [20, 22, 23] along with collaboration and participation with others [21, 22], opportunities for gaining knowledge and reciprocal assistance [22], and emotional support [25] are critical components of individual empowerment. Our analysis showed that, despite potentially disempowering healthcare system structures, communities can provide equitable access to resources that promote empowerment related skills.

Community stroke recovery groups were perceived as an important and equitable resource which supported development of empowerment related skills. These community groups also provided a space in which stroke survivors could create new social networks, and participate in learning, educational, and therapeutic opportunities. Social participation [19] and community based therapy [11], such as group exercise [19, 37] and aphasia therapy [18, 33, 37], are important aspects for stroke adaptation and recovery: particularly for the development of coping strategies and resilience, and understanding physical limitations and complex emotions [14, 33]. Collaboration and participation between members and coordinators present in these stroke recovery groups created a supportive empowerment environment. In summary, when analysed through an empowerment lens, there was evidence that some degree of empowerment was present among stroke survivors and caregivers. However, their stroke healthcare system did not appear to provide an overarching empowering environment that equitably enabled the acquisition of services necessary for optimal stroke recovery opportunities.

### Practice implications

This analysis points to the benefits of empowerment focused stroke rehabilitation as a means to improve care and support for patients [22]. Empowerment can improve coping ability, patient satisfaction, adaptation to condition, clinical outcomes, and independence and quality of life [26]. Malterud has suggested that an empowerment framework may also address inherent power imbalances associated with health professional and patient relationships [36]. Without this type of support, stroke survivors and caregivers can experience abandonment and helplessness [6]. Moreover, socioeconomic marginalization may increase their risk of health problems [36]. However, critics of empowerment healthcare models suggest that most models focus only on standard patient outcomes, such as disease management and interactions with healthcare providers [38], rather than also encompassing patients’ holistic values. Others have suggested that the concept of empowering patients can result in the transfer of social responsibility from health providers to patients [20, 24], and is used as a means to reduce healthcare costs [26]. This may be problematic and disempowering when patients do not have the ability, or skills [24], or want to take responsibility for their health [20, 24].

One potential implication for practice is the design of an empowerment framework for stroke rehabilitation and recovery policy and practise. An empowerment framework would foster holistic models of care [25, 26], enable tailoring rehabilitation to the unique and dynamic situations of stroke survivors, and open up avenues for equitable care. Additionally, this framework would support collaborative responsibility [21, 22, 25] between team members at all stages of rehabilitation, and provide opportunities for stroke survivor participation in their health care decision-making.

A second potential implication for practice is translating knowledge from empowerment orientated research into effective training programs for stroke rehabilitation health professionals. These programs would educate healthcare professionals to enact empowering and holistic approaches which should incorporate, as Bravo proposes, patients’ personal characteristics, context, and values [26]. Furthermore, such programs would provide health professionals with a richer understanding of the power imbalances in health provider patient relationship [36, 39, 40].

Empowerment theory also considers the ethical, psychosocial, and political relationships between individuals, organizations, and communities [20, 23], but few models pay attention to empowerment’s emancipatory aspect [24, 36]. A third potential implication for practice is the formation of linkages, both formal (hospital acute stroke care and IRFs) and informal (community recreational activity services such as yoga), with stroke recovery groups to support equitable stroke rehabilitation and recovery. Stroke recovery groups, which provide opportunities for support, participation, and collaboration, may fit the notion of emancipatory natural helping systems: systems in which health, wellness, equity, competence, collaboration, and adaptation are emphasised over illness and deficiency. These dimensions are fundamental components of empowerment [21, 22]. Strengthening connections between these groups, hospitals, and community healthcare may have the potential to improve outcomes for stroke survivors.

### Limitations

Qualitative research is always contextual and our findings are thus specific to the healthcare system and community resources which participants in this study experienced. In addition, empowerment is also context dependent and meanings of empowerment processes and outcomes vary: for example, according to population group, culture, and socioeconomic status [20–22].

Therefore, the findings of this study, with participants gathered from a single metropolitan city in Western Canada, may not be representative of the experiences of other population groups.

Our recruitment process limited participants to stroke recovery group attendees and our findings may not be representative of stroke survivors not attending stroke recovery groups. Seeking out and accessing stroke survivor groups may imply that stroke survivors and caregivers were already empowered. However, the data did not illuminate the relationship between being empowered and seeking out (and accessing) stroke recovery groups. Exploring how stroke survivors and caregivers seek out and access support groups may deepen understanding of the potential relationship between being empowered and seeking out and accessing support groups. Furthermore, without data from stroke survivors who did not attend stroke recovery groups, our findings were not sufficient to elucidate the extent to which being empowered enabled participation in stroke recovery groups, or the extent to which these groups contributed to stroke survivor empowerment. Further studies, which include stroke recovery group non-attendees, may draw out other layers of insight with regards to access to and the benefits of attendance at stroke recovery groups and stroke survivor empowerment.

Another limitation is the representation of specific participants. Participant recruitment was dependent on the people present at the three locations at the time of recruitment as well as their desire to participate. This resulted in a gender imbalance in stroke survivor recruitment such that our findings may not reflect a comprehensive understanding of gendered experiences of stroke recovery multidisciplinary team care. Recruitment of spousal caregivers was dependent on consent provided by both the stroke survivor and caregiver. Only one female stroke survivor was married and consent was not provided to interview her spouse. The spouses of four male stroke survivors consented to be interviewed. More research is needed to explore not only the difference in gendered stroke survivor and spousal caregiver recruitment, but also gendered experiences of stroke recovery multidisciplinary team care. Furthermore, we were unable to make comparative conclusions between stroke survivors without spousal care and those with spousal care. However, this has the potential to build on our findings and is a ripe area for future research.

With respect to the support group coordinators and speech pathologist we recruited all the individuals who coordinated the groups: 2 groups had 1 coordinator, 1 group had 2 coordinators who worked together, and one group had a speech pathologist. This small sample of support group coordinators is a reflection of the purposeful convenient sampling approach used. Further studies in other contexts may be able to recruit a more robust sample of support group coordinators and enrich our understanding of this groups’ perspective. Finally, a limitation of the demographic survey was that it did not explore underlying co-morbidities, pattern of deficits or income which may have provided deeper insight into how stroke survivors and their caregivers managed stroke. These are potential topics for future research.

Despite these limitation, our study group was heterogeneous with participants from a range of socioeconomic, ethno cultural, linguistic, and educational backgrounds. Our data reflected a range of perspectives that indicated experiences of managing stroke, while not uniformly identical, had underlying similarities. Thus, our findings may be transferred to other population groups who experience similar healthcare and community resource structures. This research contributes to the dearth of knowledge of how stroke survivors and their informal caregivers experience stroke management and how this relates to empowerment.

## Conclusion

We explored the experiences of stroke survivors, and members of their care network to identify areas in the management of stroke which could be better supported by the healthcare system and health providers. Employing an empowerment lens, our findings suggest that collaboration between patients, their healthcare providers, informal caregivers, and community resources, was lacking and fragmented in stroke rehabilitation care. In addition, many participants were unable to access rehabilitation services due to structural inequities. These various systems factors contributed to a disempowering stroke rehabilitation process and placed barriers on consistent supported rehabilitation and recovery. A holistic empowerment approach to stroke rehabilitation may address these inadequacies, as well as alleviate some of the healthcare services and social structural inequities in the stroke rehabilitation process.

## Supplementary information

**Additional file 1.** Demographic questionnaire.

**Additional file 2.** Interview guide.

## Data Availability

The datasets generated and/or analysed during the current study are not publicly available due to the personal and sensitive nature of the data and in order to protect the confidentiality of participants. This study does not have ethics approval to share the participants’ narratives publically. The datasets are available from the corresponding author on reasonable request.

## Abbreviations

IRFs: In-patient recovery facilities
