# Supplementary material for: Stroke survivors’ experiences of team support along their recovery continuum
Source: BMC Health Serv Res. 2019 Oct 21;19:723. doi: 10.1186/s12913-019-4533-z (PMC6805495; doi:10.1186/s12913-019-4533-z)
Supplement: Supplementary file 1 — Additional file 1. Demographic questionnaire. [file 12913_2019_4533_MOESM1_ESM.pdf]

a place of mind
THE UNIVERSITY OF BRITISH COLUMBIA

Negotiating Power on the Healthcare Team: Perspectives from Stroke Patients

Demographic Information

Please note that you do not have to answer each question if you do not want to

Date: _______________

Name: ____________________

Age: _______________________

Country of birth: _________________________

Language spoken at home: ___ _________________________

Languages you can read or write: ____________________________

Highest level of schooling completed: _________________________

Married/Single/Widowed/Common Law: _____________________________

Occupation (If you are retired, or unemployed please state this and list your former occupation): ____________________________

Do you volunteer anywhere? Please describe: _______________________

Number of children: ________________________

Number of grandchildren: _________________

Who lives in your household? (e.g. spouse, daughter, other caregiver, live alone): _________________________

Do you have a computer in your home that you use? ________________________ ____________________________

Do you have a health care benefits? Please explain. _________________________

When did you have your stroke?____

This questionnaire is not standardized, but is in its development

Version: June 23h, 2015
