# Supplementary material for: Stroke survivors’ experiences of team support along their recovery continuum
Source: BMC Health Serv Res. 2019 Oct 21;19:723. doi: 10.1186/s12913-019-4533-z (PMC6805495; doi:10.1186/s12913-019-4533-z)
Supplement: Supplementary file 2 — Additional file 2. Interview guide. [file 12913_2019_4533_MOESM2_ESM.pdf]

Negotiating Power on the Healthcare Team:
Perspectives from Stroke Patients

Interview Protocol

Our research team is interested the experiences and perceptions of stroke patients who are provided care by a healthcare team.

You do not have to answer any questions that make you feel uncomfortable.

Thank you for agreeing to participate in this study. Do you have any questions about the interview process? Sign consent form.

Demographic survey questionnaire

Patient Experience

1) Who is on your healthcare team (including family members, friends, etc.)?
   a. Who helps you with you recovery and care?
   b. Why do you consider them to be team members

2) What is your experience as a patient who is provided stroke care by a healthcare team?
   a. Can you describe how you and your team members work together to provide for your recovery

3) Does the care you receive focus on your personal treatment goals and wishes?
   a. Do you feel as though your preferences and treatment goals are at the centre of health care provision? Can you explain?

4) When and how do you assert yourself around your care on your healthcare team?
   a. Can you explain how you interact with your healthcare team.

(e.g. do you ask questions, do you make your own health related decisions, do you make requests etc.)?

5) Are you responsible for taking ownership over your own stroke care? What is that like for you?

Do you feel that it is up to you to determine your own stroke care?

6) Are you supported in managing your own health by members of your healthcare team? Please explain

7) Does your healthcare team communicate amongst themselves around your care?   If they do, are there ever communications problems that happen on your team?

8) Do you sometimes have to share information about your care between healthcare providers? (e.g. Are providers kept up to date about the various care you receive?)

9) Do you ever need to express yourself using the terminology and language of each healthcare provider in order to have your voice heard? Please describe.

10) What are some ways in which your care can be improved?

11) What other sources/resources do you have access to for obtaining health information? Do you ever discuss those with your healthcare providers?

Health Care Team Dynamics

10) Are there to be tensions between healthcare team members that influence the care you receive?

11) Is your voice held with value and weight in terms of how healthcare team members integrate your experience, opinions and/or suggestions?

12) Are there power dynamics on your health care team? How are these power dynamics negotiated and played out amongst team members?

13) Do you see yourself as an empowered or powerful member of your healthcare team? Please explain.

14) Who holds the most or least amount of power on your healthcare team? Please explain.

15) Do you sometimes receive conflicting treatment plans or advice from different healthcare providers? (e.g. medications that do not interact well)? What do you do when this happens?

16) If you are unsatisfied or confused about the care you receive do you have somebody on your healthcare team you can go to for help? Does this person then communicate with members of your healthcare team on your behalf?

Ask for consent to interview caregiver.
